# Effect of Radiofrequency Ablation and Comparison With Surgical Sympathectomy in Palmar Hyperhidrosis

**DOI:** 10.7759/cureus.88543

**Published:** 2025-07-22

**Authors:** Mohamed A Koura, Ibrahim Sabry Attwa

**Affiliations:** 1 Pain Management, Clemenceau Medical Center Hospital, Dubai, ARE; 2 Anesthesiology, Prime Hospitals, Dubai, ARE

**Keywords:** hds scale, hyperhidrosis, palmar, radiofrequency, sympathectomy

## Abstract

Background: Palmar hyperhidrosis is a distressing condition characterized by excessive palm sweating that significantly impacts patients' quality of life (QoL). Radiofrequency ablation (RFA) and surgical sympathectomy are effective treatment modalities. This study aims to evaluate and compare the effectiveness, patient satisfaction, and safety profiles of RFA and surgical sympathectomy in managing palmar hyperhidrosis.

Methods: This retrospective study included 91 patients diagnosed with primary palmar hyperhidrosis who underwent either RFA or surgical sympathectomy. The primary outcome was the reduction in sweating severity. Statistical analyses were performed to compare treatment outcomes between the two groups.

Results: Both RFA and surgical sympathectomy significantly reduced Hyperhidrosis Disease Severity Scale scores; however, surgical sympathectomy demonstrated greater symptom reduction at six months (p = 0.01) and 12 months (p = 0.002). Patients in the sympathectomy group reported a longer symptom relief duration (11.8 ± 3.1 vs. 9.2 ± 2.5 months, p = 0.003) and greater improvement in QoL scores at 12 months (p = 0.02). The incidence of compensatory sweating was higher in the sympathectomy group (34.0%, n = 16 vs. 18.2%, n = 8, p = 0.08), whereas recurrence of hyperhidrosis was more frequent in the RFA group (27.3%, n = 12 vs. 10.6%, n = 5, p = 0.03).

Conclusion: Both RFA and surgical sympathectomy effectively reduce sweating severity in patients with palmar hyperhidrosis. While sympathectomy provides longer-lasting symptom relief and greater QoL improvement, it is associated with a higher incidence of compensatory sweating.

## Introduction

Palmar hyperhidrosis is a distressing condition characterized by excessive sweating of the palms, significantly impacting patients' daily activities, social interactions, and psychological well-being. It results from overactivity of the sympathetic nervous system, leading to excessive stimulation of the eccrine sweat glands [1]. The condition typically manifests in childhood or adolescence and can persist throughout adulthood, causing embarrassment, emotional distress, and functional limitations in affected individuals. While conservative measures such as topical antiperspirants, iontophoresis, and botulinum toxin injections provide temporary relief, more definitive interventions are often required for long-term management [2].

Among the available treatment options, surgical sympathectomy, typically performed at the T2-T3 thoracic ganglia level, has been regarded as the gold standard for severe cases of palmar hyperhidrosis. This procedure involves the transection or clipping of the thoracic sympathetic chain, effectively reducing sweat production in the palms [3]. Despite its high success rates, surgical sympathectomy is associated with potential complications, such as compensatory hyperhidrosis, pneumothorax, and neuralgia, which may limit its widespread acceptance. As a result, less invasive alternatives have been explored to achieve comparable efficacy with fewer adverse effects [4].

Radiofrequency ablation (RFA) has emerged as a promising minimally invasive technique for the management of palmar hyperhidrosis. This procedure utilizes thermal energy to selectively ablate the sympathetic nerve fibers responsible for excessive sweating, thereby reducing palmar perspiration [5]. RFA offers several advantages, including reduced postoperative pain, shorter recovery times, and a lower risk of severe complications compared to surgical sympathectomy. However, data regarding its long-term effectiveness, recurrence rates, and patient satisfaction remain limited, necessitating further comparative studies [6]. Therefore, our study aims to evaluate and compare the effectiveness, patient satisfaction, and safety profiles of RFA and surgical sympathectomy in the management of palmar hyperhidrosis using retrospective patients' data.

## Materials and methods

Study design and setting

This retrospective cohort study was conducted at Menoufia University Hospital between May 2022 and December 2023. The study aimed to compare the clinical outcomes, symptom relief, and safety profile of RFA and surgical sympathectomy in patients with primary palmar hyperhidrosis. Medical records of patients who underwent either procedure were reviewed, and relevant demographic, clinical, and outcome data were extracted for analysis.

Population and sample size

A total of 91 patients diagnosed with primary palmar hyperhidrosis were included in the study and categorized into two groups based on the treatment received: the RFA group (n = 44) and the sympathectomy group (n = 47). Eligible participants were aged between 18 and 50 years, had a confirmed diagnosis of primary palmar hyperhidrosis with a Hyperhidrosis Disease Severity Scale (HDSS) score of 3 or 4, indicating moderate to severe disease, and had failed to achieve satisfactory symptom relief with conservative treatments such as topical or systemic antiperspirants. Patients were excluded if they had secondary hyperhidrosis due to underlying conditions such as endocrine disorders, neurological diseases, or infections; a history of previous thoracic or cervical surgical procedures; active infections or contraindications to either RFA or sympathectomy; or if they were lost to follow-up within the first 12 months post-treatment.

Study measures (radiofrequency ablation and surgical sympathectomy techniques)

RFA was performed under local anesthesia with mild sedation. Using fluoroscopic guidance, a 22-gauge radiofrequency probe with a 10-mm active tip was inserted percutaneously at the level of the T2-T3 sympathetic ganglia through a posterior paraspinal approach. The probe was positioned under anteroposterior and lateral fluoroscopic views to target the sympathetic chain adjacent to the heads of the second and third ribs. Continuous thermal radiofrequency energy was delivered at 80°C for 90 seconds per lesion. Two lesions were created on each side to improve the completeness of sympathetic disruption. The procedure was repeated bilaterally. Post-procedure monitoring was conducted to assess immediate pain, adverse events, and symptom relief.

Thoracoscopic sympathectomy was performed under general anesthesia. A single- or double-port approach was used to access the thoracic sympathetic chain. The T2-T3 ganglia were identified and either resected or coagulated using electrocautery bilaterally via video-assisted thoracoscopic surgery (VATS). All patients underwent bilateral sympathectomy to ensure effective symptom control in both palms. Pneumothorax was assessed intraoperatively, and a temporary chest drain was inserted if necessary. Patients were discharged within 24 to 48 hours postoperatively, with follow-up assessments at 1, 6, and 12 months.

Ethical considerations

The study was conducted in accordance with the Declaration of Helsinki and approved by the Institutional Ethics Committee of the Faculty of Medicine, Menoufia University, Menoufia, Egypt. Informed consent was obtained from all patients prior to treatment, and their confidentiality was maintained by anonymizing data. Due to the retrospective nature of the study, a waiver of written informed consent for data collection was granted by the ethics committee.

Statistical analysis

Statistical analysis was performed using SPSS version 28.0 for Windows (IBM Corp., Armonk, NY). Continuous variables were expressed as mean ± standard deviation (SD) and compared using an independent t-test or Mann-Whitney U test, as appropriate. Categorical variables were reported as frequencies and percentages and analyzed using the chi-square test. Changes in HDSS scores and quality-of-life (QoL) scores over time were evaluated using repeated-measures ANOVA. A p-value < 0.05 was considered statistically significant.

## Results

Baseline characteristics

The mean age of patients in the RFA and sympathectomy groups was 29.4 ± 6.2 and 30.1 ± 7.1 years, respectively (p = 0.65). There was no significant difference in the gender distribution between the two groups (50.0%; n = 22 vs. 51.1%; n = 24, p = 0.91). The mean body mass index (BMI) was comparable between the groups (23.8 ± 3.5 vs. 24.2 ± 3.8 kg/m², p = 0.58). A family history of hyperhidrosis was reported in 18 (40.9%) patients in the RFA group and 21 (44.7%) in the sympathectomy group (p = 0.72). The baseline HDSS scores were also similar (3.6 ± 0.5 vs. 3.7 ± 0.4, p = 0.48), confirming that both groups had comparable symptom severity before treatment (Table 1).

**Table 1:** Baseline characteristics of the study population Data are presented as mean ± standard deviation (SD) for continuous variables and as number (n) and percentage (%) for categorical variables. t-values correspond to independent samples t-tests, while χ² values refer to chi-square tests. A p-value < 0.05 was considered statistically significant. RFA: Radiofrequency ablation; HDSS: Hyperhidrosis Disease Severity Scale.

Characteristics	RFA group (n = 44)	Sympathectomy group (n = 47)	Test value	p-value
Age (years, mean ± SD)	29.4 ± 6.2	30.1 ± 7.1	t = 0.45	0.65
Male (%)	22 (50.0%)	24 (51.1%)	χ² = 0.01	0.91
BMI (kg/m², mean ± SD)	23.8 ± 3.5	24.2 ± 3.8	t = 0.55	0.58
Family history of hyperhidrosis (%)	18 (40.9%)	21 (44.7%)	χ² = 0.13	0.72
HDSS score (Mean ± SD)	3.6 ± 0.5	3.7 ± 0.4	t = 0.71	0.48

Primary outcome: reduction in HDSS score

At one month post-treatment, there was a reduction in HDSS scores in both groups, with the RFA group showing a mean score of 2.1 ± 0.7 and the sympathectomy group showing 1.8 ± 0.6, though the difference was not statistically significant (p = 0.09). However, at six months, the HDSS score in the sympathectomy group was significantly lower compared to the RFA group (1.6 ± 0.5 vs. 2.3 ± 0.8, p = 0.01). This trend persisted at 12 months, with the sympathectomy group maintaining a significantly lower HDSS score (1.5 ± 0.4 vs. 2.5 ± 0.9, p = 0.002), indicating superior long-term efficacy of sympathectomy in symptom reduction (Table 2).

**Table 2:** Primary outcome: reduction in hyperhidrosis disease severity scale (HDSS) score Data are presented as mean ± standard deviation (SD). A t-test was used to determine the significance. *p < 0.01 was considered highly significant, and ** indicates a highly statistically significant difference (p < 0.01). RFA: Radiofrequency ablation.

Time point	RFA group (n = 44)	Sympathectomy group (n = 47)	T-test value	p-value
Baseline	3.6 ± 0.5	3.7 ± 0.4	0.71	0.48
1 month	2.1 ± 0.7	1.8 ± 0.6	1.71	0.09
6 months	2.3 ± 0.8	1.6 ± 0.5	3.70	0.01*
12 months	2.5 ± 0.9	1.5 ± 0.4	4.15	0.002**

Secondary outcomes: symptom relief duration, quality of life, and patient satisfaction

The mean symptom relief duration was significantly longer in the sympathectomy group (11.8 ± 3.1 months) compared to the RFA group (9.2 ± 2.5 months, p = 0.003). Baseline QoL scores were similar between the groups (45.7 ± 10.4 vs. 46.1 ± 9.8, p = 0.83). However, at 12 months, patients in the sympathectomy group demonstrated significantly greater improvement in QoL scores (78.2 ± 10.1 vs. 70.5 ± 12.3, p = 0.02). Patient satisfaction was higher in the sympathectomy group, 42 (89.4%), compared to the RFA group, 34 (77.3%), but this difference was not statistically significant (p = 0.11) (Table 3).

**Table 3:** Secondary outcomes: patient satisfaction and quality-of-life improvement Data are presented as mean ± standard deviation (SD) for continuous variables and as number (n) and percentage (%) for categorical variables. *A p-value < 0.05 was considered statistically significant. **A p-value < 0.01 was considered highly statistically significant. RFA: Radiofrequency ablation.

Outcome	RFA group (n = 44)	Sympathectomy group (n = 47)	Test value	p-value
Symptom relief duration (months, mean ± SD)	9.2 ± 2.5	11.8 ± 3.1	t = 3.42	0.003**
Quality-of-life score (baseline)	45.7 ± 10.4	46.1 ± 9.8	t = 0.21	0.83
Quality-of-life score (12 months)	70.5 ± 12.3	78.2 ± 10.1	t = 2.36	0.02*
Patient satisfaction (%)	34 (77.3%)	42 (89.4%)	χ² = 2.58	0.11

Safety profile and adverse events

The incidence of compensatory sweating was higher in the sympathectomy group, 16 (34.0%), compared to the RFA group, 8 (18.2%), but the difference did not reach statistical significance (p = 0.08). Post-procedure pain was reported in six (13.6%) patients in the RFA group and 10 (21.3%) in the sympathectomy group (p = 0.32). Wound infections were observed in four (8.5%) patients in the sympathectomy group, whereas only one case (2.3%) was recorded in the RFA group (p = 0.18) (Table 4).

**Table 4:** Safety profile and adverse events Data are presented as numbers (n) and percentages (%). *A p-value < 0.05 was considered statistically significant. RFA: Radiofrequency ablation.

Adverse event	RFA group (n = 44)	Sympathectomy group (n = 47)	Test value	p-value
Compensatory sweating (%)	8 (18.2%)	16 (34.0%)	χ² = 3.14	0.08
Post-procedure pain (%)	6 (13.6%)	10 (21.3%)	χ² = 1.00	0.32
Wound infection (%)	1 (2.3%)	4 (8.5%)	χ² = 1.80	0.18
Pneumothorax (%)	0 (0%)	2 (4.3%)	χ² = 1.38	0.24
Recurrence of hyperhidrosis (%)	12 (27.3%)	5 (10.6%)	χ² = 4.70	0.03*

## Discussion

Palmar hyperhidrosis is a distressing condition characterized by excessive sweating of the palms, significantly impacting daily activities and QoL. Among the available treatments, RFA and surgical sympathectomy are widely used, yet comparative evidence regarding their efficacy, safety, and long-term outcomes remains limited [7].

Our study aimed to evaluate and compare the effectiveness, patient satisfaction, and safety profiles of RFA and surgical sympathectomy in the management of palmar hyperhidrosis. A total of 91 patients were enrolled, with 44 undergoing RFA and 47 undergoing sympathectomy. Our findings revealed that while both treatments significantly reduced hyperhidrosis symptoms, sympathectomy demonstrated superior long-term efficacy, with a greater reduction in HDSS scores at 6 and 12 months. However, sympathectomy was associated with a higher incidence of compensatory sweating and other adverse events. Despite a higher recurrence rate in the RFA group, patient satisfaction remained comparable between the two treatments. These results suggest that treatment selection should be individualized, balancing the benefits of long-term symptom relief with potential risks.

The baseline characteristics of both groups, including age, gender distribution, BMI, family history of hyperhidrosis, and HDSS scores, were comparable, ensuring that differences in treatment outcomes could be attributed to the interventions rather than confounding factors.

The primary outcome of our study was the reduction in the HDSS score over time. At baseline, both groups exhibited severe hyperhidrosis (HDSS score ≥ 3), with no significant difference between them. At one month post-procedure, both groups showed significant improvement. However, at 6 and 12 months, surgical sympathectomy demonstrated significantly lower HDSS scores compared to RFA (p = 0.01 and p = 0.002, respectively), indicating superior long-term symptom control. These findings align with previous studies such as those by Martínez-Hernández et al. and Wei et al., which reported that over 90% of patients undergoing thoracic sympathectomy experienced sustained symptom relief [8,9]. Zhu et al. also emphasized the durability of sympathectomy but noted a higher incidence of compensatory hyperhidrosis as a trade-off [10]. Conversely, RFA, while effective in the short term, was associated with a higher recurrence rate in our study (27.3% vs. 10.6%, p = 0.03), potentially due to sympathetic nerve regeneration over time - a limitation noted in prior research.

Although RFA was effective in reducing HDSS scores, the observed recurrence of symptoms at 6 and 12 months aligns with existing literature. Studies by Liu et al. also reported that RFA provides significant short-term relief, but higher recurrence rates are noted due to potential nerve regeneration [11]. The mechanism behind this recurrence is thought to be the incomplete or temporary disruption of sympathetic nerve activity, allowing partial restoration of sweating over time. Despite this limitation, RFA remains an appealing alternative due to its minimally invasive nature and reduced risk of severe complications. Hasimoto et al. highlighted that RFA has a favorable safety profile, with lower incidences of compensatory hyperhidrosis compared to sympathectomy [12].

The choice between RFA and sympathectomy must consider not only efficacy but also potential adverse effects. While we did not specifically assess compensatory hyperhidrosis, its high prevalence following sympathectomy has been well documented in previous studies, occurring in up to 60% of patients [13]. In contrast, RFA has been associated with fewer and less severe complications, making it a viable option for patients prioritizing safety over long-term efficacy. Furthermore, as reported by Nawrocki et al., patient satisfaction is often influenced not only by symptom relief but also by the presence or absence of adverse effects, suggesting that the lower complication rate of RFA may enhance patient preference despite its higher recurrence rate [14].

In terms of symptom relief duration, patients undergoing sympathectomy experienced a significantly longer mean duration of symptom control (11.8 ± 3.1 months) compared to the RFA group (9.2 ± 2.5 months, p = 0.003). This aligns with findings from Yu et al., which suggest that sympathectomy provides a more sustained effect compared to RFA, where symptom recurrence can occur earlier [15].

Hyperhidrosis significantly affects QoL, and both interventions resulted in marked improvements. At baseline, the QoL scores were comparable between the groups (p = 0.83), but at 12 months, patients in the sympathectomy group reported significantly higher scores compared to those in the RFA group (78.2 ± 10.1 vs. 70.5 ± 12.3, p = 0.02). These findings are in line with previous studies, which demonstrated that surgical sympathectomy leads to greater long-term improvements in QoL compared to less invasive modalities [14]. However, it is important to consider that while overall symptom relief and QoL improvements were greater in the sympathectomy group, patient satisfaction did not differ significantly between the two groups (89.4% vs. 77.3%, p = 0.11).

Our study demonstrated that while sympathectomy is more effective in the long term, it carries a higher risk of adverse events, particularly compensatory sweating. Compensatory sweating occurred in 34.0% of patients in the sympathectomy group compared to 18.2% in the RFA group, although the difference did not reach statistical significance (p = 0.08). Previous studies, such as Han et al., have reported compensatory hyperhidrosis rates as high as 60% following sympathectomy, reinforcing concerns about this side effect [16].

Other complications, including post-procedure pain (21.3% vs. 13.6%, p = 0.32) and wound infection (8.5% vs. 2.3%, p = 0.18), were more frequently observed in the sympathectomy group, though these differences were not statistically significant. Additionally, two cases of pneumothorax (4.3%) were reported in the sympathectomy group, whereas no such cases occurred in the RFA group (p = 0.24). These findings align with the previous work, which reported that while sympathectomy is highly effective, its invasive nature increases the risk of complications such as pneumothorax and postoperative pain [4]. In contrast, RFA presents a safer alternative with fewer severe complications, making it an attractive option for patients seeking a less invasive approach.

A key strength of our study is its direct comparison between RFA and surgical sympathectomy using objective measures, patient-reported outcomes, and safety profiles, providing valuable insights into the efficacy and risks of both treatments. Additionally, the use of standardized assessment tools enhances the reliability of our findings despite the retrospective design. However, limitations include the relatively small sample size, which may affect the generalizability of our results, and the short follow-up period, which limits our understanding of long-term recurrence and adverse effects. Future studies with larger cohorts and extended follow-up periods are necessary to confirm our findings and provide further clinical guidance.

## Conclusions

Our study highlights the effectiveness of both RFA and surgical sympathectomy in treating palmar hyperhidrosis, with sympathectomy demonstrating superior long-term symptom relief but at the cost of higher adverse event rates. RFA, while associated with a higher recurrence rate, offers a less invasive alternative with a lower risk of severe complications. Patient satisfaction remained high in both groups, suggesting that treatment decisions should be based on individual patient preferences and risk tolerance.

## References

[1] Parashar K, Adlam T, Potts G (2023). The impact of hyperhidrosis on quality of life: a review of the literature. Am J Clin Dermatol.

[2] Romero FR, Haddad GR, Miot HA, Cataneo DC (2016). Palmar hyperhidrosis: clinical, pathophysiological, diagnostic and therapeutic aspects. An Bras Dermatol.

[3] Vannucci F, Araújo JA (2017). Thoracic sympathectomy for hyperhidrosis: from surgical indications to clinical results. J Thorac Dis.

[4] Ovalı C, Sevin MB (2018). Effectiveness, success rates, and complications of different thoracoscopic sympathectomy techniques in patients with palmar hyperhidrosis. Turk Gogus Kalp Damar Cerrahisi Derg.

[5] Loscertales J, Tristán AA, Loscertales MC (2004). Thoracoscopic sympathectomy for palmar hyperhidrosis. Immediate results and postoperative quality of life. Arch Bronconeumol.

[6] Gardner PA, Ochalski PG, Moossy JJ (2008). Minimally invasive endoscopic-assisted posterior thoracic sympathectomy. Neurosurg Focus.

[7] Solish MJ, Savinova I, Weinberg MJ (2022). A practical approach to the diagnosis and treatment of palmar hyperhidrosis. Plast Reconstr Surg Glob Open.

[8] Martínez-Hernández NJ, Estors-Guerrero M, Galbis-Caravajal JM, Hervás-Marín D, Roig-Bataller A (2024). Endoscopic thoracic sympathectomy for primary hyperhidrosis: an over a decade-long follow-up on efficacy, impact, and patient satisfaction. J Thorac Dis.

[9] Wei Y, Xu ZD, Li H (2020). Quality of life after thoracic sympathectomy for palmar hyperhidrosis: a meta-analysis. Gen Thorac Cardiovasc Surg.

[10] Zhu LH, Du Q, Chen L, Yang S, Tu Y, Chen S, Chen W (2014). One-year follow-up period after transumbilical thoracic sympathectomy for hyperhidrosis: outcomes and consequences. J Thorac Cardiovasc Surg.

[11] Liu Y, Weng W, Tu Y, Wang J (2022). Chinese expert consensus on the surgical treatment of primary palmar hyperhidrosis (2021 version). Chin Med J (Engl).

[12] Hasimoto FN, Cataneo DC, Hasimoto EN, Ximenes AM, Cataneo AJ (2020). Radiofrequency in the treatment of primary hyperhidrosis: systematic review and meta-analysis. Clin Auton Res.

[13] Sang HW, Li GL, Xiong P, Zhu MC, Zhu M (2017). Optimal targeting of sympathetic chain levels for treatment of palmar hyperhidrosis: an updated systematic review. Surg Endosc.

[14] Nawrocki S, Cha J (2019). The etiology, diagnosis, and management of hyperhidrosis: a comprehensive review: therapeutic options. J Am Acad Dermatol.

[15] Yu Y, Cui J, Zhang Y, Feng L, Wang L (2024). Comparative study of CT-guided radiofrequency and alcohol ablation in the treatment of primary hyperhidrosis. Front Surg.

[16] Han Z, Rui M, Ni C, Zhu J, Xu L, Yao M (2023). The success rate and associated risk factors of CT-guided percutaneous radiofrequency sympathectomy in the treatment of primary hyperhidrosis: a retrospective observational trial. J Clin Neurosci.

